# Dopamine-β-Hydroxylase (DBH), Its Cofactors and Other Biochemical Parameters in the Serum of Neurological Patients in Bangladesh

**Published:** 2009-12

**Authors:** Md. Khalilur Rahman, Farhana Rahman, Tania Rahman, Takeshi Kato

**Affiliations:** 1 *Department of Biochemistry and Molecular Biology, University of Dhaka, Bangladesh*; 2 *Laboratory of Natural Information Science, Graduate School of Integrated Science, Yokohama City University, Japan*

**Keywords:** dopamine-β-hydroxylase, copper, ascorbic acid, zinc, neurological patients

## Abstract

Dopamine-β-hydroxylase (DBH) is a neurotransmitter synthesizing enzyme which catalyzes the formation of norepinephrine from dopamine. In this study, we measured the level of DBH activity in the serum of patients of three different age groups (8–14 yrs, 20–40 yrs and 45–60 yrs) suffering from neurological diseases. Serum DBH activity was measured in 38 neurological patients and 38 normal individuals in order to determine significant variables for its potential use to diagnose the neurological patients. It was found that the DBH activity decreased in the patients of all age groups. A considerable decrease in activity was observed in the patients of 8–14 yrs age group (14.2 nmoles/min/ml in patients compared to the normal value of 22.6). A significant decrease in activity was found in the 20–40 yrs age group (23.4 nmoles/min/ml in patients compared to the normal value of 33.0). The decrease in DBH activity was also found in the patients of 45–60 yrs age group but to a lesser extent (26.4 nmoles/min/ml in the patients compared to the normal value of 30.2). The kinetic studies of DBH exhibited an increase of K_m_ value and a decrease in V_max_ in the neurological patients. Serum copper and ascorbic acid levels (cofactors of DBH) were found to be decreased in neurological patients and hence are in agreement with the decrease in DBH activity in these patients. Other parameters such as glucose and cholesterol levels increased, protein and zinc levels decreased and ALT, AST, creatinine and urea content remained nearly unchanged in the patients’ serum.

## INTRODUCTION

Dopamine-β-hydroxylase [3, 4-dihydroxyphenylethylamine, ascorbate: oxygen oxidoreductase (3-hydroxylating), EC 1.14.17.1; abbreviated DBH] is the enzyme responsible for the biosynthesis of catecholamine neurotransmitter, noradrenaline from dopamine, ultimately leading to the formation of adrenaline by the enzyme phenylethanolamine-N-methyl transferase (PNMT) in the mammalian tissues and serum (1, 2). The product, norepinephrine, is important biochemically and pharmacologically, because these monoamines are important intracellular messenger, such as neurotransmitter and hormone and involved in the regulation of neuronal functions, behavior and emotion of higher animals. The Dopamine-β-hydroxylase (DBH) is a copper (Cu^++^) and ascorbic acid (Vit-C) dependent enzyme and thus DBH is a mixed function copper containing oxygenase (3). Enzyme activity is stimulated by the addition of dicarboxylic acids such as fumaric acid.

DBH is an intraneuronal enzyme in the sympathetic nervous system and enzyme activity was found in the adrenal medulla (4), in the brain (5) and various sympathetically innervated organs such as the heart (6, 7). In humans and laboratory animals, DBH has been measured in plasma (8) and more recently, cerebrospinal fluid (CSF) (9). The DBH in plasma originates from the sympathetic nervous system and the adrenal medulla. DBH being one of the key neurotransmitter mediating enzymes has drawn much attention from clinical and pharmacological investigators as a possible index of the sympathetic nervous functions. Deficiency of dopamine results in various neurological disorders, mainly Parkinson’s disease (10). A significant increase in DBH activity occurs in pheochromatocytoma (11).

In Bangladesh, a considerable number of people are suffering from neurological diseases and no significant research has been carried out on the effect of catecholamines and their related enzymes in neurological diseases. Therefore, the aim of this study was to compare the serum DBH activity in patients suffering from central and peripheral neurological diseases with normal individuals. In order to get a clear profile of DBH activity in neurological patients of Bangladesh, the cofactors of DBH (ascorbic acid and copper) have been measured in the serum of patients and normal individuals. In addition, other important biological parameters namely, zinc, glucose, protein, cholesterol, ALT, AST, creatinine and urea level have been estimated to establish a more detailed comparison between normal and neurological patients of Bangladesh.

## MATERIALS AND METHODS

### Sample collection

Thirty eight neurological patients of three different age groups were recruited from the neurology department of Dhaka Medical College Hospital, Bangladesh. There were twelve patients of the age group 8–14 yrs (group 1), fourteen patients of the age group 20–40 yrs (group 2) and twelve patients of the age group 45–60 yrs (group 3). The patients of group 1, 2 and 3 were as follows: group 1, epileptic and mentally retarded, group2, epileptic and early Parkinson’s disease and group 3 were Parkinson’s disease and dementia. To compare with the corresponding neurological groups, a total of thirty eight (group 1=12, group 2=14, group 3=12) healthy individuals of same three age groups were selected as normal. Blood samples were collected from all subjects at a single time point. About 5–10 ml of blood was collected from each individual by venipuncture. Blood samples were kept in an ice chamber following collection and allowed to clot. The clotted blood was centrifuged and serum was collected and stored at −20°C until use.

### Analytical method

DBH activity in human blood was measured according to the method of Nagatsu *et al* (12). The kinetics of DBH in the serum of normal and neurological patients was determined by incubating the enzyme with different concentrations of tyramine as a substrate. The concentration of ascorbic acid, a cofactor of DBH was measured by di-nitrophenyl hydrazine method modified by Lowry *et al* (13). The levels of Cu^++^ and Zn^++^ were determined using atomic absorption spectrophotometer (AAS, pye-Unicam, SP9) (14). The total protein was estimated by the method of Lowry *et al* (15). In this process, protein solution was allowed to react with Folin-Coicalteau reagent to give a colored complex which was measured spectrophotometrically. Glucose content in blood was estimated by Nelson Somogyi method (16, 17). Serum cholesterol was determined using reaction with ferric chloride (FeCl_3_) and sulfuric acid (H_2_SO_4_). Serum ALT and AST activity were determined according to the method of Retiman and Frankel (18). The creatinine content in human blood was determined by the method of Owen *et al* (19). The blood urea was determined by the Spinreact method which is an adaptation of the reaction proposed by Jung *et al* (20).The values of V_max_ (the highest velocity of the enzyme activity) and K_m_ (substrate concentration at which the enzyme activity is half of the V_max_) were determined using the Lineweaver-Burk plot (21).

### Statistical analysis

All the data were analyzed using the Statistical Package for Social Sciences (SPSS) (version 11.0 for Windows, SPSS Inc., Chicago, USA). Student t-test (two-tailed) was used to evaluate statistical differences between the two study groups. A P-value of ≤0.05 was the criterion for a statistically significant difference. Microsoft Excel and GraphPad Prism 4.0 (USA) were used for statistical analyses and graphics. Data were expressed as mean and standard deviation (±SD).

## RESULTS

### DBH activity and its cofactor content in normal and neurological patients

Table 1 shows the levels of DBH, ascorbic acid and copper in the serum of normal and patient of different age groups.

DBH activity in serum of group 1 was 22.6 ± 6.5 and 14.2 ± 4.5 nmoles/min/ml in normal and patients respectively. In group 2, these values were 33 ± 7.6 and 23.4 ± 8 nmoles/min/ml in normal and patients respectively. In group 3, the activity was 30.2 ± 5.5 and 26.4 ± 4.0 nmoles/min/ml in normal and patients respectively. For three different age groups, DBH activity was decreased considerably in patients as compared to that of normal individuals.

**Table 1:** DBH activity, its cofactor ascorbic acid and copper content in the serum of three different age groups of normal individuals and neurological patients of Bangladesh

Age groups (Years)	No. of samples	DBH activity (nmoles/min/ml of serum)		Amount of ascorbic acid (mg/dl of serum)		Amount of copper (mg/dl of serum)	
		Normal	Patient	Normal	Patient	Normal	Patient

Group 1 (8–14 yrs)	12	22.6 ± 6.5	14.2 ± 4.5^b^	1.39 ± 0.25	1.08 ± 0.30^a^	90 ± 25.2	79 ± 15.4
Group 2 (20–40 yrs)	14	33 ± 7.6	23.4 ± 8.0^b^	1.82 ± 0.80	1.18 ± 0.36^a^	131.2 ± 30.6	124.7 ± 22.5
Group 3 (45–60 yrs)	12	30.2 ± 5.5	26.4 ± 4.0	1.56 ± 0.75	1.14 ± 0.25	122 ± 19.0	115 ± 16.8

Data are presented as Mean ± SD.

a P<0.05;

b P<0.01.

The serum content of ascorbic acid, a cofactor of DBH for group 1 were 1.39 ± 0.25 and 1.08 ± 0.30 mg/dl in normal and patients respectively. In group 2, the contents of ascorbic acid were 1.82 ± 0.80 and 1.18 ± 0.36 mg/dl in normal and patients respectively. In group 3, the same were 1.56 ± 0.75 and 1.14 ± 0.25 mg/dl in normal and patients respectively. For three different age groups, ascorbic acid content was found to be decreased in patients compared to normal individuals.

In group 1, the serum contents of copper, a cofactor of DBH were 90 ± 25.2 and 79 ± 15.4 mg/dl in normal and patients respectively. In group 2, the serum contents of copper were 131.2 ± 30.6 and 124.7 ± 22.5 mg/dl in normal and patients respectively. In group 3, the same were 122 ± 19 and 115 ± 16.8 mg/dl in normal and patients respectively. For three different age groups, copper content was decreased in patient compared to normal individuals.

### The K_m_ and V_max_ values of DBH in normal and neurological patients

**Figure 1:**
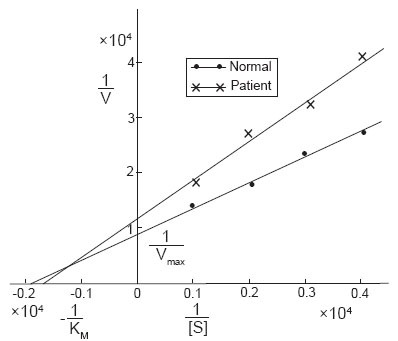
The Lineweaver-Burk plot for the determination of the K_m_ and V_max_ values of DBH in the serum of normal and neurological patients of Bangladesh.

The kinetics of DBH in the serum of normal and patients was determined using Lineweaver-Burk plot and from that plot the V_max_ and K_m_ were calculated. The K_m_ value in normal serum was found to be 5.2 × 10^−4^ and in neurological patients it was found to be 5.8 × 10^−4^. The V_max_ value in normal human serum was found to be 1.1 × 10^−4^ of serum and that in neurological patients was 0.83 × 10^−4^ of serum (Figure 1).

### Serum level of protein, glucose and cholesterol in normal and neurological patients

Table 2 shows the level of protein, glucose and cholesterol in the serum of normal and neurological patients of different age groups of Bangladesh.

**Table 2:** Protein, glucose and cholesterol content in the serum of three different age groups of normal individuals and neurological patients of Bangladesh

Age Groups (Years)	No. of Samples	Amount of total serum protein (g/dl of serum)		Amount of glucose (mg/dl of serum)		Amount of cholesterol (mg/dl of serum)	
		Normal	Patient	Normal	Patient	Normal	Patient

Group 1 (8–14 yrs)	12	6.5 ± 0.83	5.9 ±0.45^a^	91.2 ± 20.7	93.6 ± 42.0	138.62 ± 17	182.7 ±27.5^c^
Group 2 (20–40 yrs)	14	7.32 ± 0.66	6.8 ± 0.76	102.27 ± 25.1	107 ± 27.24	169.52 ± 22.35	196.13 ± 20^b^
Group 3 (45–60 yrs)	12	7.1 ± 0.8	6.3 ±0.67^a^	108.27 ± 28.6	125 ± 45.63	182.71 ± 35.11	205.24 ± 16.3

Data are presented as Mean ± SD.

a P<0.05;

b P<0.01;

c P<0.001.

The serum levels of protein of group 1 were 6.5 ± 0.83 and 5.9 ± 0.45 g/dl in normal and patients respectively. In group 2, the levels of protein were 7.32 ± 0.66 and 6.8 ± 0.76 g/dl in normal and patients respectively. In group 3, the levels were 7.1 ± 0.8 and 6.3 ± 0.67 g/dl in normal and patient respectively. For three different age groups, protein level was decreased considerably in patients compared to normal individuals.

The serum glucose levels for group 1 were 91.2 ± 20.70 and 93.6 ± 42 mg/dl in normal and patients respectively. In group 2, the serum levels of glucose were 102.27 ± 25.1 and 107 ± 27.24 mg/dl in normal and patients respectively. In group 3, the same values were 108.27 ± 28.6 and 125 ± 45.63 mg/dl in normal and patient respectively. For three different age groups, glucose levels were higher in patients compared to that of normal individuals.

In group 1, the serum cholesterol levels were 138.62 ± 17 and 182.7 ± 27.5 mg/dl in normal and patients respectively. In group 2, the levels of cholesterol were 169.52 ± 22.35 and 196.13 ± 20 mg/dl in normal and patients respectively. In group 3, the same were 182.71 ± 35.11 and 205.24 ± 16.3 mg/dl in normal and patient respectively. For three different age groups, cholesterol level was increased in patients compared to normal individuals.

### Serum level of zinc, ALT and AST in normal and neurological patients

Table 3 shows the level of zinc, ALT and AST in the serum of normal and neurological patients of different age groups in Bangladesh.

**Table 3:** Zinc, ALT and AST content in the serum of three different age groups of normal individuals and neurological patients of Bangladesh

Age groups (Years)	No. of samples	Amount of zinc (μg/dl of serum)		ALT activity (U/l of serum)		AST activity (U/l of serum)	
		Normal	Patient	Normal	Patient	Normal	Patient

Group 1 (8–14 yrs)	12	85.5 ± 12.1	75 ± 11.67^a^	14.21 ± 5	18.32 ± 7.63	16.5 ± 6.60	18.63 ± 7.4
Group 2 (20–40 yrs)	14	111.4 ± 14.34	92.6 ± 9.95^c^	17.86 ± 8.17	20.55 ± 8.68	22.7 ± 7.84	24.90 ± 14
Group 3 (45–60 yrs)	12	103 ± 10.93	90 ± 10.55^b^	20.32 ± 9.12	23.12 ± 12.3	19.58 ± 4.5	26.22 ± 12.3

Data are presented as Mean ± SD.

a P<0.05;

b P<0.01;

c P<0.001.

The zinc levels in serum of group 1 were 85.5 ± 12.10 and 75 ± 11.67 μg/dl in normal and patients respectively. In group 2, the serum zinc levels were 111.4 ± 14.34 and 92.6 ± 9.95 μg/dl in normal and patients respectively. In group 3, the values were 103 ± 10.93 and 90 ± 10.55 μg/dl in normal and patients respectively. For three different age groups, zinc level was decreased considerably in patients compared to normal individuals.

The ALT levels of group 1 were 14.21 ± 5 and 18.32 ± 7.63 U/l in normal and patients respectively. In group 2, the levels were 17.86 ± 8.17 and 20.55 ± 8.68 U/l in normal and patients respectively. In group 3, the values of ALT were 20.32 ± 9.12 and 23.12 ± 12.3 U/L in normal and patients respectively. For three different age groups, ALT level was higher in patients compared to normal individuals.

In group 1, the AST levels were 16.5 ± 6.6 and 18.63 ± 7.4 U/l in normal and patients respectively. In group 2, the values of AST were 22.7 ± 7.84 and 24.90 ± 14 U/l in normal and patients respectively. In group 3, the same were 19.58 ± 4.5 and 26.22 ± 12.3 U/l in normal and patients respectively. For three different age groups, AST level was increased in patients compared to normal individuals.

### Serum level of urea and creatinine in normal and neurological patients

Table 4 shows the levels of urea and creatinine in the serum of normal and neurological patients of different age groups of Bangladesh.

**Table 4:** Urea and creatinine content in the serum of three different age groups of normal individuals and neurological patients of Bangladesh

Age Groups (Years)	No. of Samples	Amount of urea (mmol/l of serum)		Creatinine Content (mg/dl of serum)	
		Normal	Patient	Normal	Patient

Group 1 (8–14 yrs)	12	4.8 ± 0.50	5.0 ± 0.27	0.9 ± 0.17	0.8 ± 0.16
Group 2 (20–40 yrs)	14	5.12 ± 0.42	5.2 ± 0.48	1.2 ± 0.24	0.95 ± 0.20^a^
Group 3 (45–60 yrs)	12	5.25 ± 0.49	5.4 ± 1.04	1.32 ± 0.21	1.03 ± 0.15^b^

Data are presented as Mean ± SD.

a P<0.01;

b P<0.001.

The urea content of group 1 were 4.8 ± 0.50 and 5 ± 0.27 mmol/l in normal and patients respectively. In group 2, the urea content was 5.12 ± 0.42 and 5.2 ± 0.48 mmol/l in normal and patients respectively. In group 3, the levels of urea were 5.25 ± 0.49 and 5.4 ± 1.04 mmol/l in normal and patients respectively. For three different age groups, urea level was increased considerably in patients compared to normal individuals.

The creatinine content for group 1 were 0.9 ± 0.17 and 0.8 ± 0.16 mg/dl in normal and patients respectively. In group 2, the levels of creatinine were 1.2 ± 0.24 and 0.95 ± 0.20 mg/dl in normal and patients respectively. In group 3, the same contents were 1.32 ± 0.21 and 1.03 ± 0.15 mg/dl in normal and patients respectively. For three different age groups, creatinine content was lower in patients compared to that of normal individuals.

**Figure 2:**
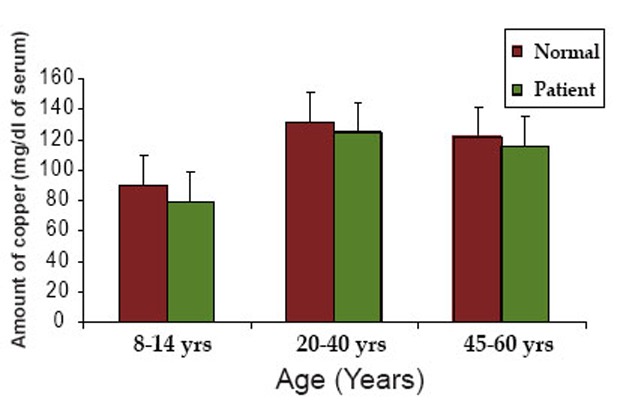
The graphical representation of the comparative levels of copper content in normal individuals and neurological patients.

**Figure 3:**
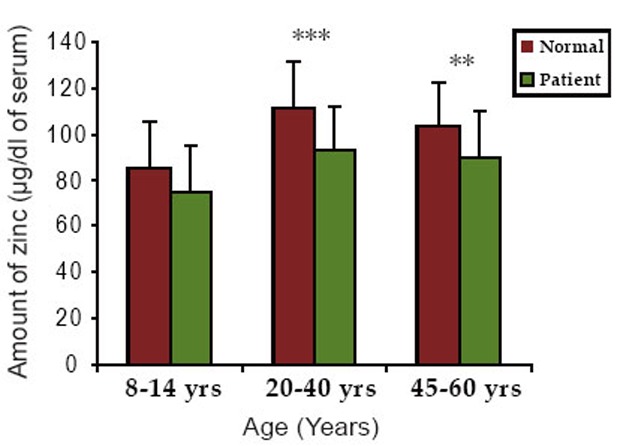
The graphical representation of the comparative levels of zinc content in normal individuals and neurological patients. *P< 0.05, **P<0.01, ***P<0.001.

Figure 2 and 3 shows the graphical representation of the comparative levels of copper and zinc content in the normal individuals and neurological patients respectively.

## DISCUSSION

Changes in DBH activity is observed in neurological disorders. Until now no systematic studies have been carried out in Bangladesh on the DBH status of neurological patients suffering from central or peripheral neurological diseases. This study fulfills this gap. A total of 38 samples from patients of three different age groups (8–14 yrs, 20–40 yrs and 45–60 yrs) were subjected to the determination of DBH activity. An equal number of samples from normal subjects were used as normal. Cofactors of DBH (Copper and ascorbic acid) and several other biochemical parameters were also determined and compared between the patients and normal subjects.

The DBH activity from these serum samples was measured by a very sensitive method proposed by Nagatsu *et al* (12). The DBH activity was found to decrease in the neurological patients of all age groups as compared to their corresponding normal groups.

A considerable decrease in DBH activity was found in the patients of 8–14 yrs age group compared to normal individual of same age group. A significant decrease in activity was found in the 20–40 yrs age group compared to that of normal. Decrease in DBH activity was also found in the patients of 45–60 yrs age group but to a lesser extent. The decrease in DBH activity in the patients indicates that there is an accumulation of dopamine and this may be responsible for causing the symptoms.

The K_m_ and V_max_ values were determined by the Lineweaver-Burk plot (21). Serum DBH of neurological patients showed a higher K_m_ and lower V_max_ compared to the normal value. Most of the neurological patients in Bangladesh have the decreased levels of dopaminergic, adrenergic and noradrenergic neurons and the decreased levels of serum DBH represent a compensatory decrease in peripheral sympathetic nerve activity. As serum DBH levels provide a useful index of sympathetic nerve functions, the decrease in DBH activity consequently lead to the decrease of noradrenaline and adrenaline in serum. Therefore, also the K_m_ value of DBH increased in the serum of neurological patients of Bangladesh. The lower V_max_ in the patient indicates a lower affinity of the enzyme for the substrate tyramine.

Copper is a cofactor of DBH enzyme and one mole of the enzyme contains two moles of cupric ions. Copper helps in maintenance in the integrity of myelin sheath (22). Serum copper content was found to be decreased considerably in the patients compared to age matched normal. The decrease in copper content in the serum of patients is in accordance with the decrease in DBH activity.

Ascorbic acid a coenzyme of dopamine-β-hydroxylase, is a powerful reducing agent and it readily gives up an electron to convert Fe^3+^ into Fe^2+^. This conversion is critical in getting sufficient iron absorbed to prevent anemia. In this study it was found that the serum ascorbic acid content decreased in the patients as compared to the normal group. This decrease may support the decrease in DBH activity. However, it has been found that enzyme activity is unaffected by even severe ascorbic acid deficiency. So, the specific role of ascorbic acid as a cofactor of DBH *in vivo* is not clear.

Zinc has a beneficial effect on the process of tissue repair and wound healing. Zinc content was found to decrease in the patients as compared to age matched normal. This finding is in agreement with the finding of Ivore *et al* that dopamine levels are significantly higher in zinc deficient animals (23). This suggests the behavioral changes in zinc deficient animals may be in part catecholamine related. Furthermore Gordon *et al* and Hesse *et al* showed that reduced zinc is an indication of increased emotional (fear) activity in animals (24, 25).

Among other biological components measured were protein, glucose and cholesterol. The total protein concentration was found to be slightly lower in the patients as compared to normal groups. The glucose concentration in the serum of the patients was slightly increased as compared to age matched normal but did not vary significantly. The cholesterol level increased in patients as compared to the normal group.

Measurements of the concentration in serum of ALT and AST can give important information regarding the severity of damage to the heart and liver diseases. In our study, the serum ALT and AST levels were found to be within the normal range. This indicates that the experimental subjects had no heart or liver damage.

Increase in levels of blood creatinine and urea give evidence of marked impairment of kidney function. In terminal stages of chronic nephritis and in some cases of acute nephritis marked urea retention may occur. In our study the creatinine and urea levels did not vary markedly in the patients and normal group. This indicates that none of the patients had damaged renal function.

From the results of our study we can conclude that the DBH activity was found to be lowered in neurological patients. The enzyme kinetic studies are in agreement with this finding. The levels of ascorbic acid, copper (cofactors of DBH) and zinc were found to be decreased in patients. Other parameters such as glucose and cholesterol levels increased, protein levels decreased and ALT, AST, creatinine and urea did not change significantly.

Since DBH activity decreases in neurological disorders, it may be useful as one of the parameters to diagnose such disorders. It is important to find out whether the decrease is due to a decrease in the substrate dopamine or other factors.
